# Comparative Transcriptome Analysis Identifies Genes Putatively Involved in 20-Hydroxyecdysone Biosynthesis in *Cyanotis arachnoidea*

**DOI:** 10.3390/ijms19071885

**Published:** 2018-06-27

**Authors:** Xiu Yun Lei, Jing Xia, Jian Wen Wang, Li Ping Zheng

**Affiliations:** 1 College of Pharmaceutical Sciences, Soochow University, Suzhou 215123, China; 18862239302@163.com (X.Y.L.); jxia@stu.suda.edu.cn (J.X.); jwwang@suda.edu.cn (J.W.W.); 2 Department of Horticultural Sciences, Soochow University, Suzhou 215123, China

**Keywords:** *Cyanotis arachnoidea*, roots, leaves, transcriptome, 20-hydroxyecdysone, biosynthesis

## Abstract

*Cyanotis arachnoidea* contains a rich array of phytoecdysteroids, including 20-hydroxyecdysone (20E), which displays important agrochemical, medicinal, and pharmacological effects. To date, the biosynthetic pathway of 20E, especially the downstream pathway, remains largely unknown. To identify candidate genes involved in 20E biosynthesis, the comparative transcriptome of *C. arachnoidea* leaf and root was constructed. In total, 86.5 million clean reads were obtained and assembled into 79,835 unigenes, of which 39,425 unigenes were successfully annotated. The expression levels of 2427 unigenes were up-regualted in roots with a higher accumulation of 20E. Further assignments with Kyoto Encyclopedia of Genes and Genomes (KEGG) pathways identified 49 unigenes referring to the phytoecdysteroid backbone biosynthesis (including 15 mevalonate pathway genes, 15 non-mevalonate pathway genes, and 19 genes for the biosynthesis from farnesyl pyrophosphate to cholesterol). Moreover, higher expression levels of mevalonate pathway genes in roots of *C. arachniodea* were confirmed by real-time quantitative PCR. Twenty unigenes encoding CYP450s were identified to be new candidate genes for the bioreaction from cholesterol to 20E. In addition, 90 transcription factors highly expressed in the roots and 15,315 unigenes containing 19,158 simple sequence repeats (SSRs) were identified. The transcriptome data of our study provides a valuable resource for the understanding of 20E biosynthesis in *C. arachnoidea*.

## 1. Introduction

*Cyanotis arachnoidea* C. B. Clarke (Commelinaceae), a Chinese traditional medicine herb, has been use for treatment of limb numbness and rheumatoid arthritis, as well as for promoting blood circulation and muscle relaxing [1]. Rich phytoecdysteroids, including 20-hydroxyecdysone (20E), dihydroxyrubrosterone, rubrosterone, poststerone, and cyanosterone B, were found in this plant [1,2]. 20E was reported to have agricultural applications in enhancing the synchronous development of *Bombyx mori*, elevating silk yield [3] and reducing the time of the molting cycle of *Alpheus heterochelis* [4]. 20E and its derivatives exhibited a wide variety of pharmacological effects, including anti-depression, antioxidation, anti-diabetes, and neuron protection [5,6]. 20E is primarily obtained from ecdysteroid-rich plants, such as *C. arachnoidea*, *Ajuga turkestanica*, and *Serratula wolffi* [6]. Dried roots and aerial parts from *C. arachnoidea* have been reported to contain 20E at 5.50% and 0.52%, respectively [7]. However, due to the limitation of the wild resources for *C. arachnoidea*, the supply of 20E is in great shortage and its application is always restricted [6].

Originally, the ecdysteroid biosynthetic pathway has been described as a cytosolic pathway, starting from mevalonate (MVA) as a precursor [8]. Subsequently, it has been proven that isopentenyl diphosphate (IPP) and dimethylallyl diphosphate (DMAPP) required for the triterpenoid synthesis can be produced not only from the MVA pathway in cytosol but also the 2-*C*-methyl-d-erythritol 4-phosphate (MEP) pathway in plastids [9]. However, the studies to date on phytoecdysteroid biosynthesis are limited to the parts mediated by the MVA pathway [8]. In many plants, ecdysteroids were synthesized from cholesterol [10,11]. However, Adler and Grebenok [12] identified lathosterol as a precursor of 20E in spinach. It is obviously controversial whether cholesterol or lathosterol is the preferred substrate for phytoecdysteriod biosynthesis [8]. Conversion of a sterol to ecdysteroid requires several structural modifications, such as the hydroxylation of the sterol nucleus and side chain [12]. In insects, cytochrome P-450 monooxygenases (CYP450s), which catalyze the bioreaction from cholesterol to 20E, have been cloned and identified. However, only one CYP450 enzyme in plants has been identified to be involved in 20E biosynthesis in the hairy roots of *Ajuga reptans* [13]. To date, the biosynthesis of 20E, especially the origin of the precursor IPP and its downstream pathway, remains largely unknown.

The accumulation of 20E in *C. arachnoidea* was different among various tissues. Mu et al. [7] reported that the 20E content in roots was 4–20 times higher than that in leaves. Biosynthesis and accumulation of secondary metabolites are often tissue-specific [14]. Tomás et al. [6] reported that the ecdysteroid content was related to organized structures of whole plants and in vitro propagated plantlets in *A. reptans*, while calluses from leaves or roots could not produce ecdysteroids [15]. The cultured plantlets of *A. reptans* produced seven ecdysteroids with the tissue differentiation. Tomas et al. has revealed that ecdysteroid production is root-specific in *A. reptans* [16]. In recent years, comparative transcriptome analysis has been applied to investigate the biosynthesis of secondary metabolites. Yang et al. compared the transcriptome of leaves and roots from *Salvia miltiorrhiza* and identified candidate genes involved in the biosynthesis of tanshinones [17]. Biosynthesis of withanolide A, a medicinal component synthesized specifically in roots of *Withania somnifrea*, was also investigated by comparative transcriptome analysis [18]. Despite the important pharmacological value of 20E, there is little genetic information revealing the biosynthesis of 20E in *C. arachnoidea*. Thus, as a follow-up to our previous works on the biotechnological production of 20E [19] and cloning of key enzyme genes in 20E biosynthesis [20], we conducted a comparative transcriptome analysis of the leaves and roots of *C. arachnoidea* to identify genes putatively involved in 20E biosynthesis. As we know, this study reported the de novo sequencing of *C. arachnoidea* for the first time. Meanwhile, transcription factors (TFs), which have been found to regulate the secondary metabolism in plants, were also searched for in the transcriptome database. Furthermore, we provided valuable information for developing the important molecular marker of simple sequence repeats (SSRs). This study was designed to characterize the transcriptome of *C. arachnoidea* and provide a valuable basis for further elucidating the biosynthetic pathway of 20E.

## 2. Results and Discussion

### 2.1. Quantification of 20E from C. arachnoidea

**Figure 1:**
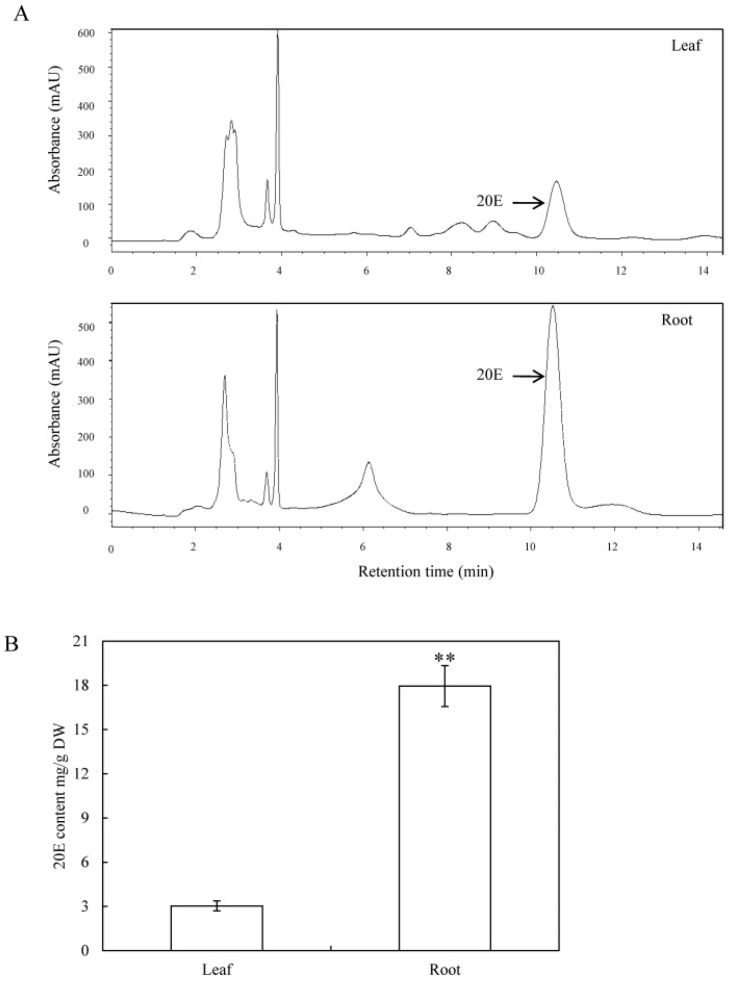
(**A**) Typical high-performance liquid chromatography (HPLC) chromatograms and (**B**) 20E contents in leaves and roots of *C. arachnoidea*. 20E was isolated from four-month old *C. arachnoidea*. Data are expressed as means ± standard deviation of three replicates. Statistical significance in comparison with the corresponding control values is indicated by ** *p* < 0.01.

As shown in Figure 1, 20E was found in both *C. arachnoidea* leaves and roots at different levels. The 20E contents were much (i.e., 6-fold) higher in roots (18.0 mg/g DW) than in leaves (3.0 mg/g DW). It has been reported that the ecdysteroid content varies in different organs of the plants [6]. 20E content in roots of *C. arachnoidea* was reported to be 4–20 times higher than that in leaves [7]. Zhu et al. [21] demonstrated that the 20E content of *C. arachnoidea* roots was the highest in air-dried whole plants, amounting to 2.9% (*w*/*w*). Although 20E production is not root-specific in *C. arachnoidea*, such spatial distribution of 20E suggests a root preferential expression pattern of biosynthetic pathway genes.

### 2.2. Library Sequencing and De Novo Assembly and Annotation

To determine the transcriptome of *C. arachnoidea* leaves and roots, two cDNA libraries were established and sequenced using a HiSeq™2500 platform (Illumina, San Diego, CA, USA). Finally, 45,368,044 and 41,095,316 clean reads were obtained from leaves and roots, respectively (Table 1). We generated a unique transcript library for *C. arachnoidea* using both sets of reads and obtained a total of 79,835 unigenes with an average length of 894 bp and a *N*_50_ length of 1268 bp. The length distribution of unigenes was presented in Figure S1. Clean reads from leaves and roots were separately mapped to the *C. arachnoidea* transcript library. Finally, 69,782 and 70,556 unigenes were obtained for the leaves and roots, respectively (Table 1).

For sequence annotation, BLASTX alignment was performed against non-redundant (NR), Swiss-Prot, Gene Ontology (GO) and Kyoto Encyclopedia of Genes and Genomes (KEGG) databases. As a result (Figure S2), 39,057 (48.92%) unigenes received gene descriptions through comparisons with known proteins in the NR database, and 49.55% of annotated unigenes exhibited strong homology (*E* value < 1.0 × e^−60^). Most of these annotated unigenes (30.23%) were best matched to sequences from *Musa acuminata* subsp. Malaccensis, followed by *Elaeis guineensis* (19.74%), and *Phoenix doctylifera* (16.04%). A sum of 28,791 unigenes (36.06%) was annotated using the Swiss-Prot database. Additionally, 27,111 (33.96%) and 9922 (12.43%) unigenes were annotated using the GO and KEGG databases, respectively. Figure S3a showed the number of unigenes annotated by all the databases.

### 2.3. Different Expression Genes (DEGs) Identified Between Leaves and Roots

The transcript abundance of each unigene was calculated through the fragments per kilobase per million reads (FPKM) method and those with |log_2_ FoldChange| ≥ 1, with a *p* value ≤ 0.05, were considered as DEGs. Finally, a sum of 4811 DEGs was identified in which 2427 unigenes were up-regulated while 2384 unigenes were down-regulated in *C. arachnoidea* roots (Table S2). In addition, we identified 1180 DEGs which were only expressed in leaves and 1250 DEGs expressed specifically in roots (Figure S3b).

### 2.4. Identification of Unigenes Involved in Secondary Metabolism

The biological function and pathway assignment of unigenes were conducted through KEGG pathway annotation. In the end, 9922 (12.43%) unigenes were mapped to five categories (i.e., organismal systems, metabolism, genetic information processing, environmental information processing, and cellular processes) and 32 sub-categories (Figure S4). The higher proportion of unigenes belonged to the pathway of signal transduction, carbohydrate metabolism, and translation. The majority of unigenes were classified into the metabolism category, and the number of unigenes related to different secondary metabolisms was listed in Table 2. Out of all unigenes involved in secondary metabolite pathways, unigenes in “Phenylpropanoid biosynthesis (ko00940)” had the highest proportion, followed by “Stilbenoid, diarylheptanoid, and gingerol biosynthesis (ko00945)”, “Terpenoid backbone biosynthesis (ko00900)”, and “Flavonoid biosynthesis (ko00941)”. Additionally, 59 unigenes were mapped to the “Steroid biosynthesis (ko00100)” pathway (Figure S5). Among 1070 unigenes related to secondary metabolism, 192 unigenes were differentially expressed in *C. arachnoidea* leaves and roots.

### 2.5. Identification of Unigenes Related to 20E Biosynthesis

**Figure 2:**
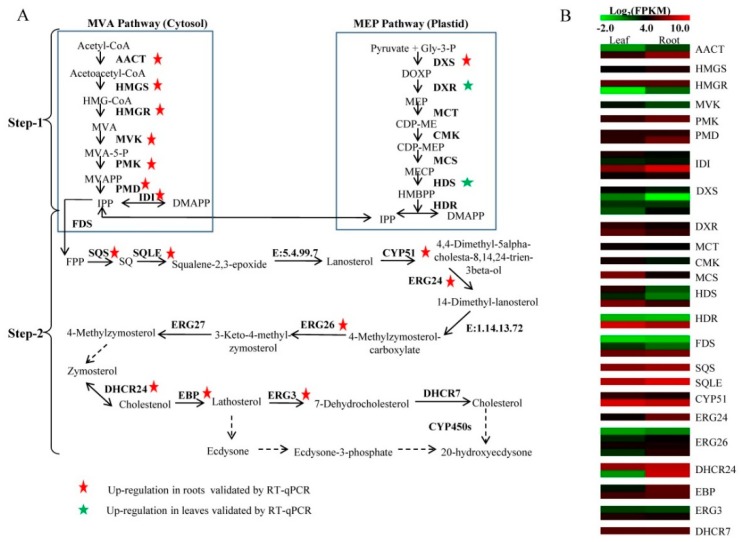
(**A**) The putative pathway of 20E biosynthesis in *C. arachnoidea*. (**B**) The gene expression was validated by RT-qPCR. The solid arrows denote known steps while the dashed arrows denote multi-step and incomplete reactions. Enzymes related to the different steps are shown between the reactions catalyzed. The expression level of unigenes related to these enzymes in leaf and root is shown by a heatmap. Names of the enzymes are provided in Table 3.

**Figure 3:**
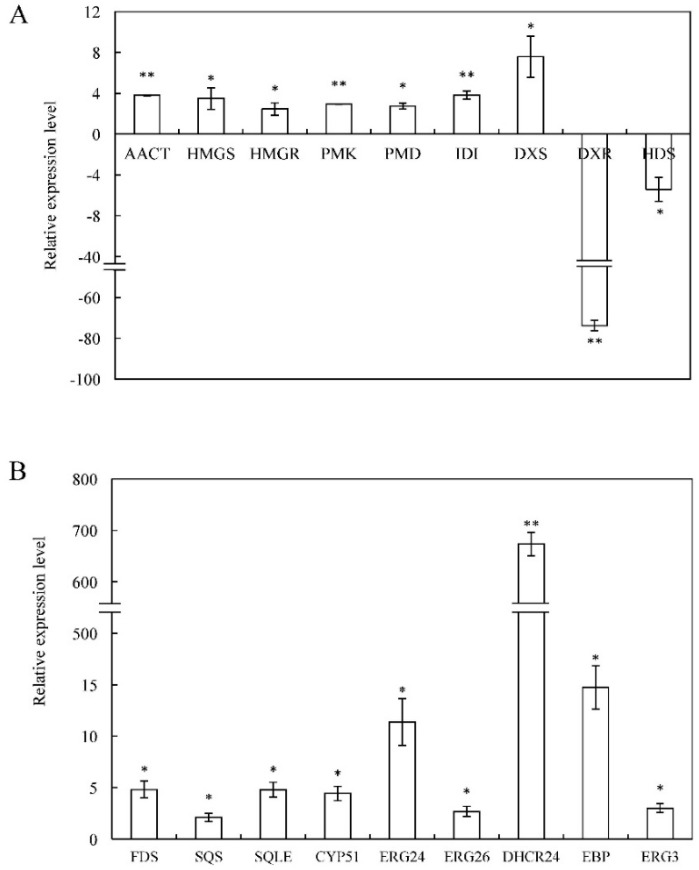
RT-qPCR analysis of selected genes involved in 20E biosynthesis. (**A**) Unigenes involved in terpenoid backbone biosynthesis. (**B**) Unigenes involved in steroid biosynthesis. Leaves and roots of four-month old *C. arachnoidea* were collected and their total RNAs were used for RT-qPCR analysis. Relative expression level of unigenes in roots was calculated by the 2^−ΔΔ*C*t^ method with actin as an internal control and was compared to those in leaves, which were all set to be 1. The negative value represents higher expression levels of unigenes in leaves than in roots. The error bar indicated standard deviations of three biological replicates. Asterisks represented significant differences: * *p* < 0.05 and ** *p* < 0.01. The full names of the abbreviations (unigenes) are provided in Table 3.

20E is an isoprenoid-derived compound using five-carbon building units of IPP or its possible isomer, DMAPP, which can be synthesized by both the MVA and MEP pathway in plants. Previous reports showed that phytoecdysteroid biosynthesis is limited to parts mediated via the MVA pathway [8]. In the *C. arachnoidea* transcriptome, unigenes encoding all the known structural enzymes in the upstream pathways up to IPP were found, including 11 unigenes for six enzymes in the MVA pathway, 15 unigenes for seven enzymes in the MEP pathway, and four unigenes for isopentenyl diphosphate isomerase (IDI) (Step 1 in Table 3 and Figure 2). According to the FPKM value, unigenes involved in the MVA pathway mostly showed higher expression levels in *C. arachnoidea* roots, such as *AACT* (comp70319_c0_seq8), *PMK* (comp65552_c0_seq7), and *PMD* (comp59477_c0_seq1). On the contrary, unigenes involved in the MEP pathway had higher expression level in leaves, such as *DXR* (comp47417_c0_seq1, comp74054_c0_seq1), *MCS* (comp30840_c0_seq1), *HDS* (comp33211_c0_seq1, comp67980_c0_seq1), and *HDR* (comp73401_c0_seq1). Moreover, the expression profile of unigenes involved in the MVA and MEP pathways were validated through real-time quantitative PCR (RT-qPCR) (Figure 3). Unigenes with the |relative expression level| ≥ 2 were presented in Figure 3A. The expression level of *IDI* was up-regulated 3.82-fold in roots. Expression of *ACTT* was up-regulated in roots by 3.80-fold. Additionally, unigenes encoding HMGR, HMGS, PKM, and PMD involved in the MVA pathway all had relative higher expression levels in *C. arachniodea* roots than in leaves. Except for DXS, unigenes encoding DXR and HDS involved in the MEP pathway were down-regulated in *C. arachnoidea* roots (Figure 3A). Because 20E is highly accumulated in roots, the genes encoding enzymes involved in 20E biosynthesis are expected to show a root-preferential expression pattern. These results implied that the backbone of 20E could be synthesized mainly through the MVA pathway rather than the MEP pathway in roots.

In many plants, ecdysteroids are synthesized from the C27 sterol cholesterol [22]. Additionally, lathosterol was also proved as a precursor for ecdysteroid biosynthesis [12]. The result of the pathway assignment mentioned above showed that 59 unigenes were mapped to the “Steroid biosynthesis (ko00100)” pathway. According to the map showed in Figure S5, the downstream steps of 20E biosynthesis were presented in Step 2 of Figure 2. Unigenes encoding most of the enzymes involved in lathosterol and cholesterol biosynthesis were found in our transcriptome database (Step 2 in Table 3). These unigenes showed higher expression levels in roots compared to leaves. For example, *SQLE* (comp35298_c0_seq1), *ERG24* (comp52043_c0_seq5), *DHCR24* (comp29143_c0_seq1, comp48248_c0_seq1), and *EBP* (comp47084_c0_seq3) all had higher FPKM values in roots. To validate the expression level of these unigenes, RT-qPCR analysis was conducted. As shown in Figure 3B, the expression level of *DHCR24* was 673.45-fold higher in roots than in leaves. The expression of *EBP* and *ERG24* was up-regulated in roots by 14.8- and 11.4-fold, respectively. In all, we identified 49 unigenes encoding 24 enzymes involved in 20E biosynthesis in *C. arachniodea*.

### 2.6. Validation of Differentially Expressed CYP450s

Conversion of a sterol to ecdysteroid requires several structural modifications, such as the hydroxylation of the sterol nucleus and side chain [12]. In insects, the biosynthesis of 20E from cholesterol was catalyzed by CYP450 enzymes [23]. The hydroxylation steps in plants are presumably mediated by CYP450s as they are in insects [12]. Tsukagoshi et al. [13] identified a CYP450 enzyme CYP71D443, which catalyzes the C-22 hydroxylation of 20E in *Ajuga* hairy roots. There was only one reported CYP450 enzyme to be involved in the 20E biosynthesis of plants so far [13]. In order to identify the possible involvement of CYP450s in 20E biosynthesis, up-regulated CYP450s unigenes in roots were selected from DEGs. As a result, 20 unigenes encoding CYP450s were identified and their up-regulated expression levels in roots were validated using RT-qPCR (Table 4). For example, the expression level of unigene comp36620_c0_seq1 was 1666.05-fold higher in roots than in leaves. The expression level of comp67837_c0_seq4 in roots was 597.69-fold higher than in leaves. Much work needs be done to narrow down these candidate CYP450 genes to reveal the genes responsible for the bioreaction from cholesterol to 20E.

### 2.7. Transcription Factors Predicted and Statistics of Simple Sequence Repeats 

In our research, TFs were searched for in the *C. arachnoidea* transcriptome, and a total of 1312 TFs were found (Table 5). Among all the TFs identified, basic helix-loop-helix (bHLH), ethylene response factor (ERF), and C2H2 -type zinc finger family members were of a higher proportion. Furthermore, 90 TFs were up-regulated while 58 TFs were down-regulated in *C. arachnoidea* roots. Among the up-regulated TFs, MYBranked the highest (15), followed by bHLH (13), and WRKY (10). In plants, TFs have been found to regulate secondary metabolism pathways [14]. For example, an ERF transcription factor named JRE4 stimulates the biosynthesis of steroidal glycoalkaloids (SGAs), cholesterol-derived metabolites, through activating the transcription of SGA biosynthetic genes, such as *HMGS*, *HMGR*, *IDI*, and *SQS* [24]. Considering the root-preferential accumulation of 20E, these up-regulated TFs were worth further investigating for their function in 20E biosynthesis.

SSRs are one of the most important molecular markers and have various applications in genetics and plant breeding [25]. Thus, SSRs in *C. arachnoidea* were analyzed in our present study. Out of 79,835 unigenes, 15,315 unigenes containing 19,158 SSRs were identified (Table 6). Within these SSRs, 1194 SSRs presented in a compound formation. Mononucleotide repeats were the most abundant (14,598), followed by tri-nucleotide repeats (2270), and di-nucleotide repeats (2100). Penta-nucleotide repeats were the least (17). We also checked the SSRs in unigenes involved in 20E biosynthesis. Unigenes comp68996_c0_seq2 (*HMGR*) and comp63129_c0_seq5 (*ERG26*) contained compound SSRs. Comp71987_c0_seq5 encoding *DXR* had a di-nucleotide repeat. Additionally, the eight unigenes encoding AACT, HMGS, DXS, DXR, MCT, HDS, DHCR24, and EBP contained mononucleotide repeats. SSRs at different positions in a gene can help determine the regulation of gene expression and the function of the protein produced [25]. Sharopova [26] reported that genes containing five or more SSRs had the highest average level of expression in *Arabidopsis*. In rice, amylase content was correlated with a variation in the number of SSRs in the 5’-untranslated region of the waxy gene [27]. These SSRs identified in the *C. arachnoidea* transcriptome were valuable genetic resources for future studies of this species.

## 3. Materials and Methods

### 3.1. Plant Materials

*C. arachnoidea* seeds were collected in September 2011 from the suburbs of Luquan County, Yunnan Province of China with its voucher specimen (SCU-110923), identified by C.Y. Liu. They were deposited in the herbarium of Soochow University. Germination and transplantation were performed in the horticultural nursery of Soochow University, Suzhou, China. One month after germination, the seedlings were transplanted into a plastic pot (35 × 25 × 7 cm in length, width, and height, respectively) containing sand and vermiculite in a 1:2 (*v*/*v*) ratio. Cultures were maintained in a growth chamber at 25 ± 2 °C under a 14/10-h light/dark cycle photoperiod with white fluorescent light at 1500 lux. Leaf and root tissues of four-month old *C. arachnildea* were collected separately, frozen in liquid nitrogen, and stored at −80 °C until use. At least three biological replicates were used for subsequent studies, and each replicate contained leaf or root tissues from at least 15 seedlings.

### 3.2. 20E Extraction and Analysis

The extraction and quantification of 20E in *C. arachnoidea* leaves and roots were conducted as described by our previous report [20] with slight modifications. Dry leaves or roots (0.5 g) were ground into a powder and extracted with 30 mL methanol under sonication for 90 min. The extract was then evaporated to dryness and dissolved in 1 mL methanol. High performance liquid chromatography (HPLC) conditions are as follows: an Aglient 1280 HPLC system equipped with 250 × 4.6 mm Aglient HC-C18 column, samples were eluted with 20:80 (*v*/*v*) acetonitrile/water at a flow rate 1 mL/min, and monitored at 242 nm. 20E was quantified with a genuine standard (Sigma, St. Louis, CA, USA). Figure 1A presented a typical chromatogram of 20E in *C. arachnoidea* leaves and roots under the condition.

### 3.3. cDNA Library Construction, Sequencing and Quality Control

For cDNA library construction, total RNA was firstly extracted from leaf or root tissues of four-month old *C. arachnildea* using a *mir*Vana™ RNA isolation kit (Applied Biosystems, Foster City, CA, USA) and then treated with DNase I for 30 min at 37 °C. RNA integrity and purity was confirmed using the Agilent 2100 Bioanalyzer (Agilent Technologies, Palo Alto, CA, USA). For each pool, total RNA (10 μg) was prepared for the cDNA library. The mRNA was purified from total RNA using magnetic beads with Oligo (dT). Subsequently, the mRNA was sheared into small fragments for cDNA synthesis. Double-stranded cDNA was synthesized using random hexamers. These cDNA fragments were subjected to an end repair process, the ligation of adapters, and were enriched by PCR to create the final libraries. Paired-end sequencing at 125 bp was performed using a HiSeq™2500 platform (Illumina, San Diego, CA, USA). High-quality reads were obtained by removing adaptor fragments, reads containing more than 5% ambiguous bases, and low-quality reads containing more than 20% of bases with a *Q* value ≤ 20.

### 3.4. De Novo Assembly and Sequence Annotation

De novo assembly of all clean reads was performed using the Trinity program (version: trinityrnseq_r20131110). The raw RNA-seq data were submitted to NCBI’s Gene Expression Omnibus (GEO) repository under accession number SRP144398 (http://www.ncbi.nlm.nih.gov). Further processing to form longer sequences was undertaken using the software TGICL (http://compbio.dfci.harvard.edu/tgi/software/) and, finally, the unigenes were acquired. All unigenes were assigned a putative gene description following BLASTX alignment to the NR (ftp://ftp.ncbi.nih.gov/blast/db), Swiss-Prot (http://www.uniprot.org/downloads), GO (http://www.geneontology.org/), and KEGG (http://www.genome.jp/kegg/pathway.html) databases, with a cut off *E* value of ≤ 1 × e^−5^. To gain an overview of gene pathway networks, all unigenes were mapped to KEGG pathways using the KEGG Automatic Annotation Server. The number of unigenes corresponding to different KEGG pathways was calculated.

### 3.5. Identification of DEGs

Clean reads from *C. arachnoidea* leaves and roots were separately mapped to the assembled transcripts, and the transcript abundance of each unigene was calculated using the FPKM method. Bowtie2 (http://bowtie-bio.sourceforge.net/bowtie2/manual.shtml) and eXpress (http://www.rna-seqblog.com/express-a-tool-for-quantification-of-rna-seq-data/) were used for mapping the sequencing reads to calculate the FPKM values. In this work, the significance of gene expression differences was assessed using the |log_2_ FoldChange| ≥ 1 and a *p* value ≤ 0.05. 

### 3.6. Real-Time Quantitative PCR Analysis

The expression levels of selected unigenes were analyzed through RT-qPCR. Total RNA of *C. arachnoidea* leaves and roots were isolated with the RNAprep Pure Plant Kit (Tiangen, Beijing, China). The first cDNA strand was synthesized using the RevertAid First Strand cDNA Synthesis Kit (Thermo Scientific, San Jose, CA, USA) according to the manufacturer’s instructions. RT-qPCR was performed using the CFX96™ Real-Time System (Bio-Red, Hercules, CA, USA). The reaction mixture included 2 μL five-fold diluted cDNA template, 1 μL of 10 mM forward primer, 1 μL of 10 mM reverse primer, 10 μL FS Universal SYBR Green Master (Roche, Indianapolis, IN, USA), and 6 μL ddH_2_O. Amplification conditions were 94 °C for 4 min, and then 40 cycles of 94 °C for 1 min, 56 °C for 30 s, and 72 °C for 15 s. All the primers used were listed in Table S1.

### 3.7. Identification of Simple Sequence Repeats

The identification of SSRs in the *C. arachnoidea* transcriptome was conducted using a microsatellite program (MISA) (http://pgrc.ipk-gatersleben.de/misa/). SSRs from the mononucleotides to the hexa-nucleotides were searched for in all unigenes. Both perfect and compound repeats were identified.

### 3.8. Statistical Analysis

To examine significant differences statistically between the means of two groups, we used Microsoft Excel software to conduct Student’s *t*-test. Values are reported as the mean ± SD from three independent experiments. Asterisks represented significant differences: * *p* < 0.05 and ** *p* < 0.01.

## 4. Conclusions

Our research established the transcriptome database of the important medicinal plant, *C. arachniodea*, for the first time. In total, 79,835 unigenes were assembled and 39,425 unigenes were successfully annotated. The comparative analysis of the *C. arachniodea* leaf and root transcriptome demonstrated that the expression levels of 2427 unigenes were up-regulated in roots with a higher accumulation of 20E. Forty-nine unigenes encoding enzymes in the MVA and MEP pathways and in the downstream of phytoecdysteroid backbone biosynthesis were identified. The higher expression levels of MVA pathway genes in roots of *C. arachniodea* implied that 20E biosynthesis could be mainly mediated by the MVA pathway. Moreover, twenty unigenes encoding CYP450s were identified to be the new candidate genes for the bioreaction from cholesterol to 20E. In addition, 90 TFs up-regulated in roots and molecular marker SSRs were analyzed for further research on gene regulation and plant breeding. Our work will be helpful in understanding phytoecdysteroid biosynthesis and providing important genetic information on *C. arachniodea*.

## Figures and Tables

**Table 1:** Summary of the sequencing data of the leaf and root transcriptome of *C. arachnoidea*.

The Sequencing Data	Leaf	Root
Clean bases	5,671,005,500	5,136,914,500
Clean reads	45,368,044	41,095,316
Q30 (%)	90.63	89.90
GC content (%)	43.50	44.50
Unigenes (≥ 300 bp)	69,782	70,556
All unigenes (≥ 300 bp)	79,835	
*N*_50_ (bp)	1268	
Average length (bp)	894	

**Table 2:** Numbers of unigene related to the secondary metabolism biosynthetic pathway in *C. arachnoidea*.

Secondary Metabolites Biosynthesis Pathway	Total	Up-Regulated Unigenes	Down-Regulated Unigenes
Phenylpropanoid biosynthesis (ko00940)	289	62	17
Stilbenoid, diarylheptanoid, and gingerol biosynthesis (ko00945)	85	9	5
Terpenoid backbone biosynthesis (ko00900)	79	1	1
Flavonoid biosynthesis (ko00941)	79	2	12
Limonene and pinene degradation (ko00903)	68	4	8
Ubiquinone and other terpenoid-quinone biosynthesis (ko00130)	67	4	12
Isoquinoline alkaloid biosynthesis (ko00950)	63	8	2
Steroid biosynthesis (ko00100)	59	4	2
Carotenoid biosynthesis (ko00906)	43	1	6
Tropane, piperidine and pyridine alkaloid biosynthesis (ko00960)	39	1	2
Brassinosteroid biosynthesis (ko00905)	37	7	0
Diterpenoid biosynthesis (ko00904)	35	3	2
Zeatin biosynthesis (ko00908)	34	3	1
Flavone and flavonol biosynthesis (ko00944)	24	0	1
Isoflavonoid biosynthesis (ko00943)	16	0	1
Monoterpenoid biosynthesis (ko00902)	14	1	4
Sesquiterpenoid and triterpenoid biosynthesis (ko00909)	12	3	1
Anthocyanin biosynthesis (ko00942)	8	0	0
Betalain biosynthesis (ko00965)	6	0	0
Geraniol degradation (ko00281)	5	0	0
Glucosinolate biosynthesis (ko00966)	4	1	0
Caffeine metabolism (ko00232)	2	0	0
Polyketide sugar unit biosynthesis (ko00523)	1	0	0
Indole alkaloid biosynthesis (ko00901)	1	1	0
Total	1070	115	77

The transcript abundance of each unigene was calculated through the FPKM method, and those with |log_2_ FoldChange| ≥ 1, with a p value ≤ 0.05, were considered as DEGs. Positive and negative log_2_ FoldChange values indicates up- and down-regulated unigenes, respectively.

**Table 3:** The FPKM value of unigenes which encode enzymes involved in 20E biosynthesis.

Enzymes	Enzyme Commission Number	Unigenes ID	FPKM Value	
			Leaf	Root
**Step 1: MVA pathway**				
acetyl CoA acetyltransferase (AACT)	2.3.1.9	comp60224_c0_seq7	1.35	4.97
		comp70319_c0_seq8	36.31	114.60
3-hydroxy-3-methyl-glutaryl coenzyme A synthase (HMGS)	2.3.3.10	comp69323_c0_seq6	15.40	26.53
3-hydroxy-3-methyl-glutaryl coenzyme A reductase (HMGR)	1.1.1.34	comp68996_c0_seq2	59.92	55.43
		comp52491_c0_seq3	0.29	2.98
		comp31434_c0_seq1	0	1.82
mevalonate kinase (MVK)	2.7.1.36	comp69732_c0_seq13	10.58	5.18
		comp120320_c0_seq1	0	1.10
phosphomevalonate kinase (PMK)	2.7.4.2	comp65552_c0_seq7	35.22	69.05
diphosphomevalonate decarboxylase (PMD)	4.1.1.33	comp33484_c0_seq1	29.43	28.81
		comp59477_c0_seq1	30.56	72.50
**MEP pathway**				
1-deoxy-d-xylulose-5-phosphate synthase (DXS)	2.2.1.7	comp66526_c0_seq5	17.96	9.56
		comp24131_c0_seq1	2.04	0.25
		comp71989_c1_seq3	6.86	6.63
		comp59095_c0_seq1	3.92	12.75
1-deoxy-d-xylulose-5-phosphate reductoisomerase (DXR)	1.1.1.267	comp47417_c0_seq1	41.16	0
		comp71987_c0_seq5	31.46	22.10
		comp74054_c0_seq1	59.57	35.26
2-*C*-methyl-d-erythritol4-phosphate cytidylyi transferase (MCT)	2.7.7.60	comp71928_c2_seq77	21.02	15.51
4-diphosphocytidyl-2-*C*-methyl-d-erythritol kinase (CMK)	2.7.1.148	comp74100_c0_seq1	24.09	10.73
2-*C*-methyl-d-erythritol-2,4-cyclodiphosphate synthase (MCS)	4.6.1.12	comp30840_c0_seq1	88.79	22.58
hydroxy-2-methyl-2-(E)-butenyl 4-diphosphate synthase (HDS)	1.17.7.1	comp33211_c0_seq1	30.23	4.26
		comp53197_c0_seq1	7.95	2.19
		comp67980_c0_seq1	91.56	39.75
hydroxy-2-methyl-2-(E)-butenyl 4-diphosphate reductase (HDR)	1.17.1.2	comp90900_c0_seq1	0.82	0.69
		comp73401_c0_seq1	331.58	174.81
isopentenyl diphosphate isomerase (IDI)	5.3.3.2	comp64868_c0_seq4	24.51	13.51
		comp61897_c0_seq2	8.51	10.42
		comp29765_c0_seq1	106.24	337.84
		comp74567_c0_seq1	19.86	25.58
**Step 2**				
farnesyl diphosphate synthase (FDS)	2.5.1.10	comp96291_c0_seq1	0.47	0.57
		comp60714_c0_seq9	6.56	3.03
		comp28732_c0_seq1	71.68	87.98
squalene synthase (SQS)	2.5.1.21	comp68019_c0_seq1	105.92	144.18
squalene monooxygenase (SQLE)	1.14.13.132	comp35298_c0_seq1	201.88	387.62
sterol 14-demethylase (CYP51)	1.14.13.72	comp67692_c0_seq1	33.34	24.69
		comp67692_c1_seq3	335.08	250.89
δ14-sterol reductase (ERG24)	1.3.1.70	comp52043_c0_seq5	21.53	69.73
sterol-4α-carboxylate 3-dehydrogenase (ERG26)	1.1.1.170	comp40656_c0_seq1	1.25	1.91
		comp63129_c0_seq5	8.84	18.50
		comp48599_c0_seq1	22.32	23.31
		comp69306_c0_seq26	7.47	29.19
δ24-sterol reductase (DHCR24)	1.3.1.72	comp29143_c0_seq1	136.31	215.60
		comp48248_c0_seq1	1.45	272.54
cholestenol δ-isomerase (EBP)	5.3.3.5	comp47084_c0_seq3	10.87	59.19
		comp60181_c0_seq1	40.63	54.68
δ7-sterol 5-desaturase (ERG3)	1.14.19.20	comp50441_c0_seq1	5.89	5.25
		comp61289_c0_seq4	28.21	24.24
7-dehydrocholesterol reductase (DHCR7)	1.3.1.21	comp71639_c2_seq1	55.04	56.70

**Table 4:** Validation of the expression levels of CYP450 unigenes by using RT-qPCR

Unigene ID	Description	Foldchange	RT-qPCR
comp36620_c0_seq1	cytochrome P450 94C1-like	Inf	(1.67 ± 0.13) × 10^3^ **
comp56632_c0_seq1	cytochrome P450 90B1-like	Inf	38.29 ± 10.74 *
comp60467_c1_seq1	cytochrome P450 710A1-like	Inf	49.69 ± 0.24 **
comp70580_c0_seq1	cytochrome P450 704C1	Inf	32.05 ± 7.01 *
comp28108_c0_seq1	cytochrome P450 CYP736A12	1013.73	(1.26 ± 0.27) × 10^2^ *
comp30002_c0_seq1	cytochrome P450 90A1 isoform X2	388.30	(1.83 ± 0.63) × 10^2^
comp67837_c0_seq4	cytochrome P450 86B1-like isoform X2	147.93	(5.98 ± 0.71) × 10^2^ **
comp57563_c0_seq2	cytochrome P450 71A1-like	124.00	19.39 ± 1.42 **
comp61518_c0_seq1	cytochrome P450 90A1-like	115.05	(4.67 ± 0.34) × 10^2^ **
comp71681_c0_seq1	cytochrome P450 71A1-like	101.70	(4.15 ± 0.10) × 10^2^ **
comp69723_c0_seq2	cytochrome P450 86B1-like	82.30	43.66 ± 8.29 *
comp60312_c0_seq1	cytochrome P450 734A1-like	56.02	(1.12 ± 0.07) × 10^2^ **
comp60108_c0_seq3	cytochrome P450 90B1-like	47.42	73.56 ± 3.60 **
comp60517_c0_seq1	cytochrome P450 734A6-like	39.25	2.10 ± 0.19 **
comp66356_c0_seq1	cytochrome P450 71A1-like	33.79	18.72 ± 2.78 **
comp70580_c1_seq4	cytochrome P450 704C1-like	22.36	24.68 ± 3.93 **
comp60316_c0_seq2	cytochrome P450 714B3-like	21.86	33.65 ± 4.75 **
comp66583_c0_seq3	cytochrome P450 714D1-like	19.16	19.30 ± 7.73
comp67012_c0_seq1	cytochrome P450 734A6-like	16.32	17.48 ± 4.38 **
comp70760_c4_seq11	Cytochrome P450 86A1	12.32	(2.99 ± 0.07) × 10^2^ **

Foldchange equals the ratio of S1/S2. S1 means the FPKM value of a unigene in roots while S2 means the FPKM value of a unigene in leaves. RT-qPCR represents the relative expression level of unigenes in roots measured by RT-qPCR compared to those in leaves. Values are reported as mean ± SD from three independent experiments. Asterisks represented significant differences: * *p* < 0.05 and ** *p* < 0.01.

**Table 5:** Summary of transcription factor (TF) unigenes in *C. arachnoidea*

TF Family	Number of Unigenes Detected	Up-Regulated in Roots	Down-Regulated in Roots
bHLH	146	13	10
ERF	80	3	1
C2H2	79	6	3
NAC	78	4	4
WRKY	75	10	5
MYB-related	74	8	4
bZIP	63	7	0
G2-like	60	4	2
MYB	60	15	1
HD-ZIP	49	2	7
Others	548	18	21
Total	1312	90	58

The transcript abundance of each unigene was calculated through the FPKM method, and those with |log_2_ FoldChange| ≥ 1 with a *p* value ≤ 0.05 were considered as DEGs. Positive and negative log_2_ FoldChange values indicates up- and down-regulated unigenes, respectively.

**Table 6:** Statistics of simple sequence repeats (SSRs) identified from *C. arachnoidea* transcriptome data.

SSR Statistics	Number
Total number of sequences examined	79,835
Total size of examined sequences (bp)	71,342,686
Total number of identified SSRs	19,158
Number of SSR containing sequences	15,313
Sequences containing more than 1 SSR	3067
SSRs present in compound formation	1194
Mononucleotide repeats	14,598
Di-nucleotide repeat	2100
Tri-nucleotide repeat	2270
Tetra-nucleotide repeat	155
Penta-nucleotide repeat	17
Hexa-nucleotide repeat	18

## Supplementary Materials

The following are available online at http://www.mdpi.com/1422-0067/19/7/1885/s1.
