# Supplementary material for: Comparative Transcriptome Analysis Identifies Genes Putatively Involved in 20-Hydroxyecdysone Biosynthesis in Cyanotis arachnoidea
Source: Int J Mol Sci. 2018 Jun 27;19(7):1885. doi: 10.3390/ijms19071885 (PMC6073978; doi:10.3390/ijms19071885)
Supplement: Supplementary file 1 [file ijms-19-01885-s001.zip › Table S1 and Figures.pdf]

Supplementary Materials: Comparative Transcriptome Analysis Identifies Genes Putatively Involved in 20-Hydroxyecdysone Biosynthesis in *Cyanotis arachnoidea*

Xiu Yun Lei [1], Jing Xia [1], Jian Wen Wang [1] and Li Ping Zheng [2,*]

[1] College of Pharmaceutical Sciences, Soochow University, Suzhou 215123, China; 18862239302@163.com (X.Y.L.); jxia@stu.suda.edu.cn (J.X.); jwwang@suda.edu.cn (J.W.W)

[2] Department of Horticultural Sciences, Soochow University, Suzhou 215123, China

* Correspondence: lpzheng@suda.edu.cn; Tel.: +86-521-65880195

Figure S1: Size distribution of assembled unigenes in the *C. arachnoidea* transcriptome.

Figure S2: Species and *E*-value distributions of BLASTX data. (a) Distribution among species of BLASTX unigene matches made against the NR protein database (cutoff *E* value ≤ 1.0 × e^{-5}) and the percentage assigned of each species. Different colors represent different species in the right-hand pie chart. (b) *E*-value distributions of unigenes giving top BLAST hits in the NR database.

Figure S3: (a) Venn diagram of annotated unigenes using different databases and (b) different expression genes (DEGs) between leaf and root samples.

Figure S4: KEGG classification of all unigenes. The ordinate is the name of the pathway, and the abscissa is the proportion of unigenes belonging to this pathway. Unigenes were divided into five branches: cellular processes, environment information processing, genetic information processing, metabolism, and organismal systems.

Figure S5: Map of the steroid biosynthesis pathway generated by KEGG analysis. Green boxes denoted unigenes that were identified in the pathway.

Table S1: List of primers used for the validation of expression through RT-qPCR.

Table S2: List of DEGs between leaves and roots of *C. arachnoidea*.

Table S1. List of primers used for validation of expression through RT-qPCR.

Unigene ID	Description	Forward primers (5'-3')	Reverse primers (5'-3')
actin	Reference gene	AGGCCGTCCTCTCCCTTTAT	GCGGTCGTCGTGAAAGAGTA
comp36620_c0_seq1	cytochrome P450 94C1-like	AGCAGGGTCGACAAGAACTG	CCTCAATCCATCTCTCCGGC
comp56632_c0_seq1	cytochrome P450 90B1-like	CGGCGTATCGAAAAGCTGTG	GCGAAAGAGCAATAGCACCG
comp60467_c1_seq1	cytochrome P450 710A1-like	CCTTTGACCTCCCTGGCTTT	ATCTCTGGGAGTGCGTTGTG
comp70580_c0_seq1	cytochrome P450 704C1	ACCACAAGGCTATGCTCACT	ACTTGAAGCATCATCCAGCTCA
comp28108_c0_seq1	cytochrome P450 CYP736A12	TTGGCGGGAAGCTTAGTGAG	TCCCCTCCACGTCCCTTAAT
comp30002_c0_seq1	cytochrome P450 90A1 isoform X2	CAGCTACCCGACCTCCATC	GGCCAGGCGATTGATAGTCAT
comp67837_c0_seq4	cytochrome P450 86B1-like isoform X2	GATCGATCCGGGGTGTCTTC	CGTCCAAGTACCTCAACCCC
comp57563_c0_seq2	cytochrome P450 71A1-like	ATGTTTAGCGCTGGAACCGA	GGCGTGATTCGATCTTTGCC
comp61518_c0_seq1	cytochrome P450 90A1-like	TCGATCTACAGGAGCACGGA	CCAATCCAAGGCCTCGTTCT
comp71681_c0_seq1	cytochrome P450 71A1-like	GCACTCGCCCCTTTTTAAGC	CACGTGTGATCCTCTCCGTC
comp69723_c0_seq2	cytochrome P450 86B1-like	CATGTCGTTCAATTCGGGGC	GAGAGCTTAGGCTCGACGAC
comp60312_c0_seq1	cytochrome P450 734A1-like	AGCTATGGGGCGAAAATGCT	TCCAATGCACACTCTAGGGC
comp60108_c0_seq3	cytochrome P450 90B1-like	TCCTGGCGCAGATTTAGCAA	GTAAACCCCCATGGAAGCCA
comp60517_c0_seq1	cytochrome P450 734A6-like	TTGGTTTAGTGGCCTTGGGG	GTTTTGTGGCACCATTGGCT
comp66356_c0_seq1	cytochrome P450 71A1-like	TCCCTCATCGTTCCCTAGCA	CTTGGTGTGGGGATGAGACC
comp70580_c1_seq4	cytochrome P450 704C1-like	CTATCCGCCGATTGCTGGAA	ATGGCTCAACTCTCCCTTGC
comp60316_c0_seq2	cytochrome P450 714B3-like	CGCTCGGTGGGATTCACATA	GAACTCATGCACGTCTTCGC
comp66583_c0_seq3	cytochrome P450 714D1-like	GGGACATAGGCCAGCACTTT	TCGCCCAAATAATGGGCCTT
comp67012_c0_seq1	cytochrome P450 734A6-like	GAGCGAAGGTGGACGTAGAG	TCGGGACCCCAAAACTTAGC
comp70760_c4_seq11	Cytochrome P450 86A1	CGTCGAGAGAGCAATCGTCA	CATCGGCGACGACGTACTTA
comp70319_c0_seq8	acetyl-CoA acetyltransferase (AACT)	CATCACGTTTTTCGCCGTCA	CGTGCAACACCAACAACACA
comp69323_c0_seq6	hydroxymethylglutaryl-CoA synthase (HMGS)	TCAGAAAGCGCGAAGGATG	CCCTTGCTTGCCCCATCATA
comp68996_c0_seq2	3-hydroxy-3-methylglutaryl coenzyme A reductase (HMGR)	CCATCACTTGAGGTGGGGAC	ACCAGCAACGATGGTAGCAA
comp65552_c0_seq7	phosphomevalonate kinase (PMK)	CAATGCAGCTGGGGTTCCTA	ACTGAGTCGCTTGCATCTCC
comp33484_c0_seq1	mevalonate diphosphate decarboxylase (PMD)	TCCACCACAATCGGAAACTGA	CTCCCAACTGTGGTGCAGAT

comp29765_c0_seq1	isopentenyl-diphosphate Delta-isomerase I (IDI)	CCAGAGGCAGCTGACATGAA	ACATTCGACGAGCTCAAGCA
comp59095_c0_seq1	1-deoxy-D-xylulose-5-phosphate synthase (DXS)	CAGGTTTCATTGCCCACTGC	TCACGGAGCTTGCGTAGTTT
comp47417_c0_seq1	1-deoxy-D-xylulose 5-phosphate reductoisomerase (DXR)	GGGACAAGCCCAAGCCTATT	CTGATCAGCTAAGAGCGCGA
comp33211_c0_seq1	4-hydroxy-3-methylbut-2-en-1-yl diphosphate synthase (HDS)	TGGATGATGCAACTGATGCC	CTGCACGATGTCTGCACAAAA
comp28732_c0_seq1	farnesyl pyrophosphate synthase (FDS)	CGGAGTTGCTCCAAGATCCA	AACAACTGACAGTCCGCGAT
comp68019_c0_seq1	squalene synthase (SQS)	TCGAAAGCGTTTGGAGGGAA	CCACAAGACAGCCGATCAGA
comp35298_c0_seq1	squalene monooxygenase (SQLE)	CCAACGGTGCTGAAATCCAC	AAATTGCAGCGACGAGAACG
comp67692_c1_seq3	obtusifoliol 14-alpha demethylase (CYP51)	TGACTTTGCTGCCACTCCAA	ACAACAGATAACCCGCAGCA
comp52043_c0_seq5	delta(14)-sterol reductase (ERG24)	TTCGTGGACTTCTGTCGTCG	CAGCTACAACCGTAGGCGAT
comp48599_c0_seq1	3-beta-hydroxysteroid-dehydrogenase (ERG26)	CCTAGGAAGCGGGAAAGTGG	AGCTTAGCCATTGCCGTGAT
comp48248_c0_seq1	delta(24)-sterol reductase (DHCR24)	CGGATGCGTCGTCAGATGTA	CTACCCGTCATAAGGACCGC
comp47084_c0_seq3	3-beta-hydroxysteroid-Delta(8),Delta(7)-isomerase (EBP)	ACTCCGCGATTGTGAGTGTT	TTTCGTTGCAATCGCGTACC
comp50441_c0_seq1	delta(7)-sterol-C5(6)-desaturase (ERG3)	TTGGTTGGGCAGCCTATGTT	TGAGTTGCGTGGAGATGCTT

Figure S1

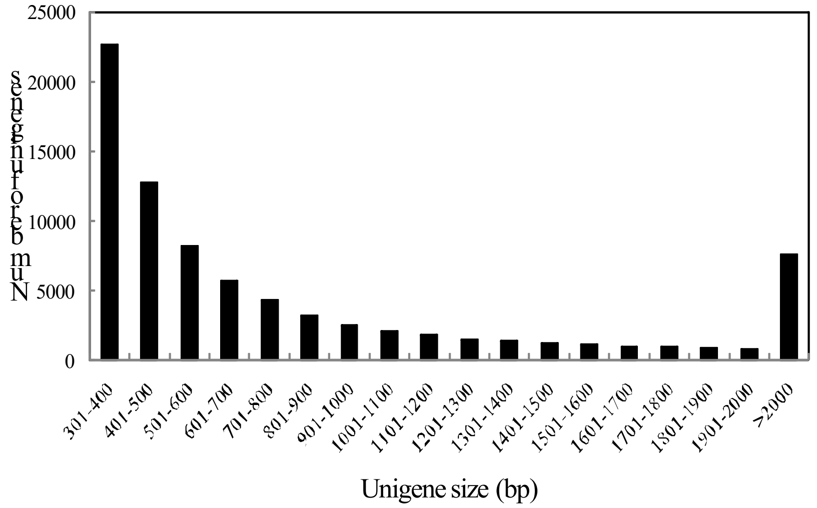

Figure S1. Size distribution of assembled unigenes in *C. arachnoidea* transcriptome.

Figure S2

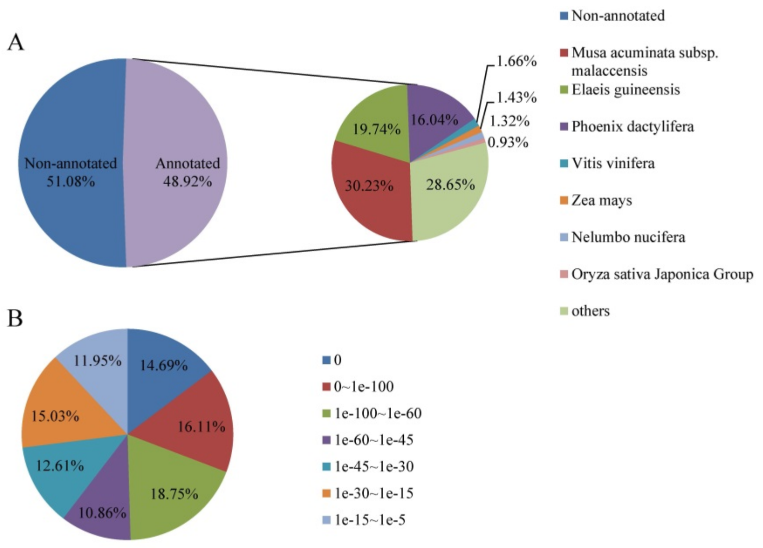

Figure S2. Species and E-value distributions of BLASTX data. (A) Distribution among species of BLASTX unigenes matches made against the NR protein database (cutoff *E* value ≤1.0 e^{-5}), and the percentage assigned of each species. Different colors represent different species in the right-hand pie chart. (B) *E*-value distributions of unigenes giving top BLAST hits in the NR database.

Figure S3

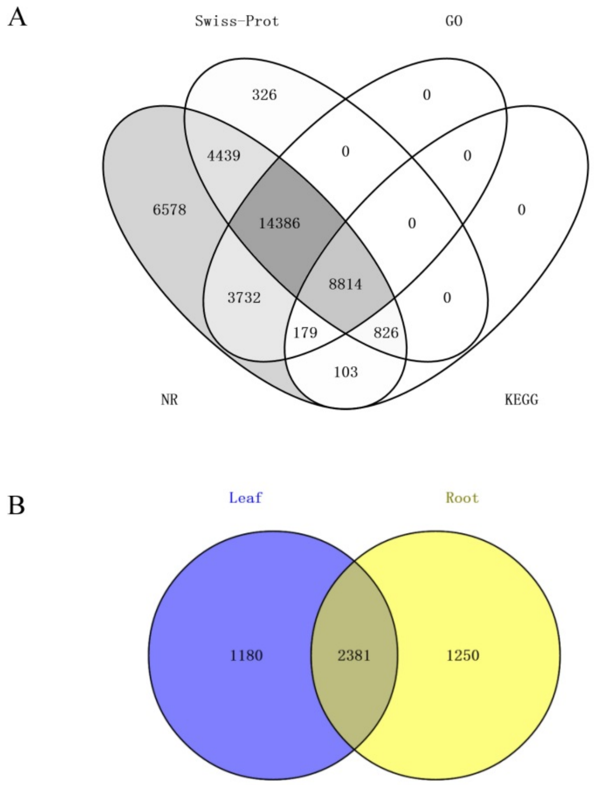

Figure S3. Venn diagram of annotated unigenes using different databases (A) and different expression genes (DEGs) between leaf and root samples (B).

Figure S4

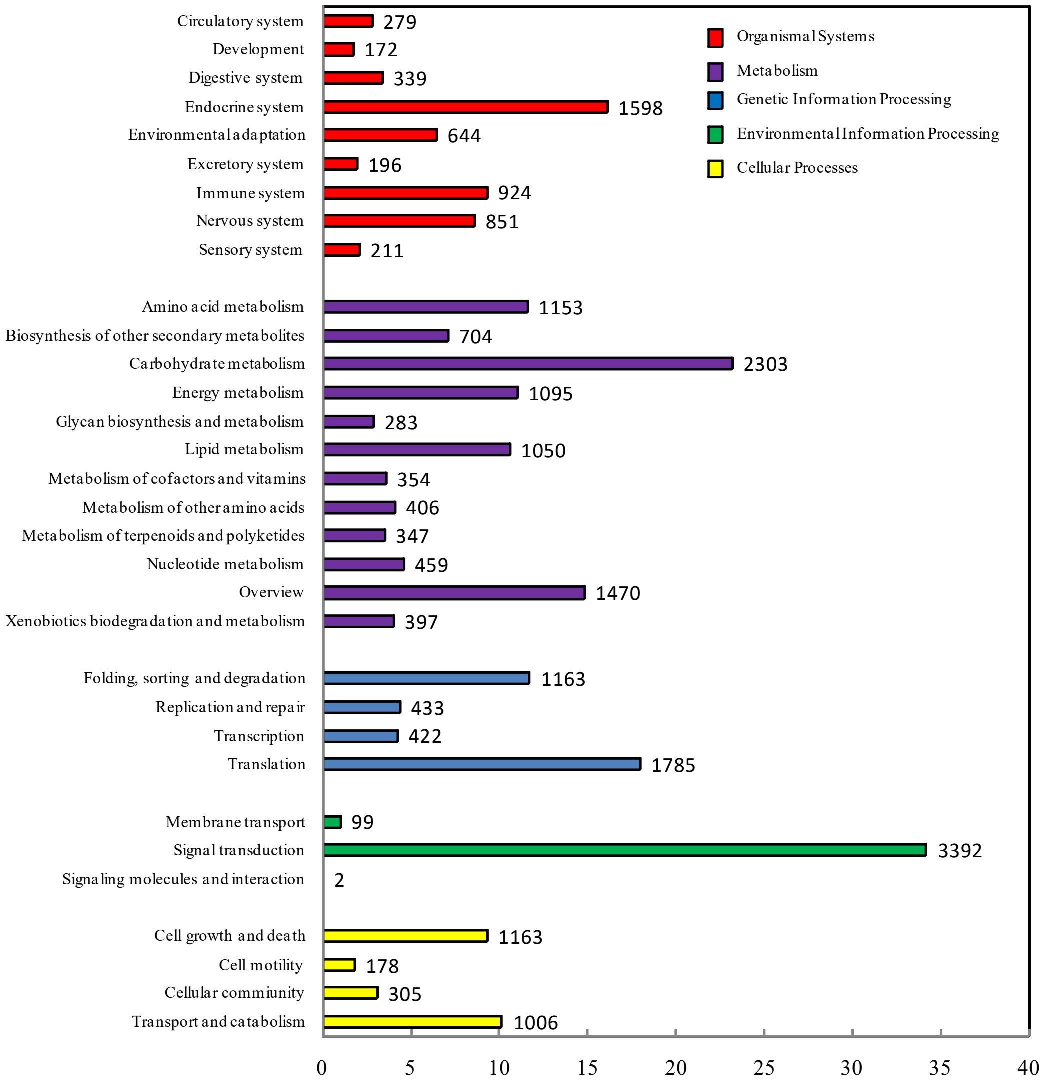

Figure S4. KEGG classification of all unigenes. The ordinate is the name of the pathway and the abscissa is the proportion of unigenes belonging to this pathway. Unigenes were divided into five branches: Cellular Processes; Environment Information Processing; Genetic Information Processing; Metabolism; Organismal Systems.

Figure S5

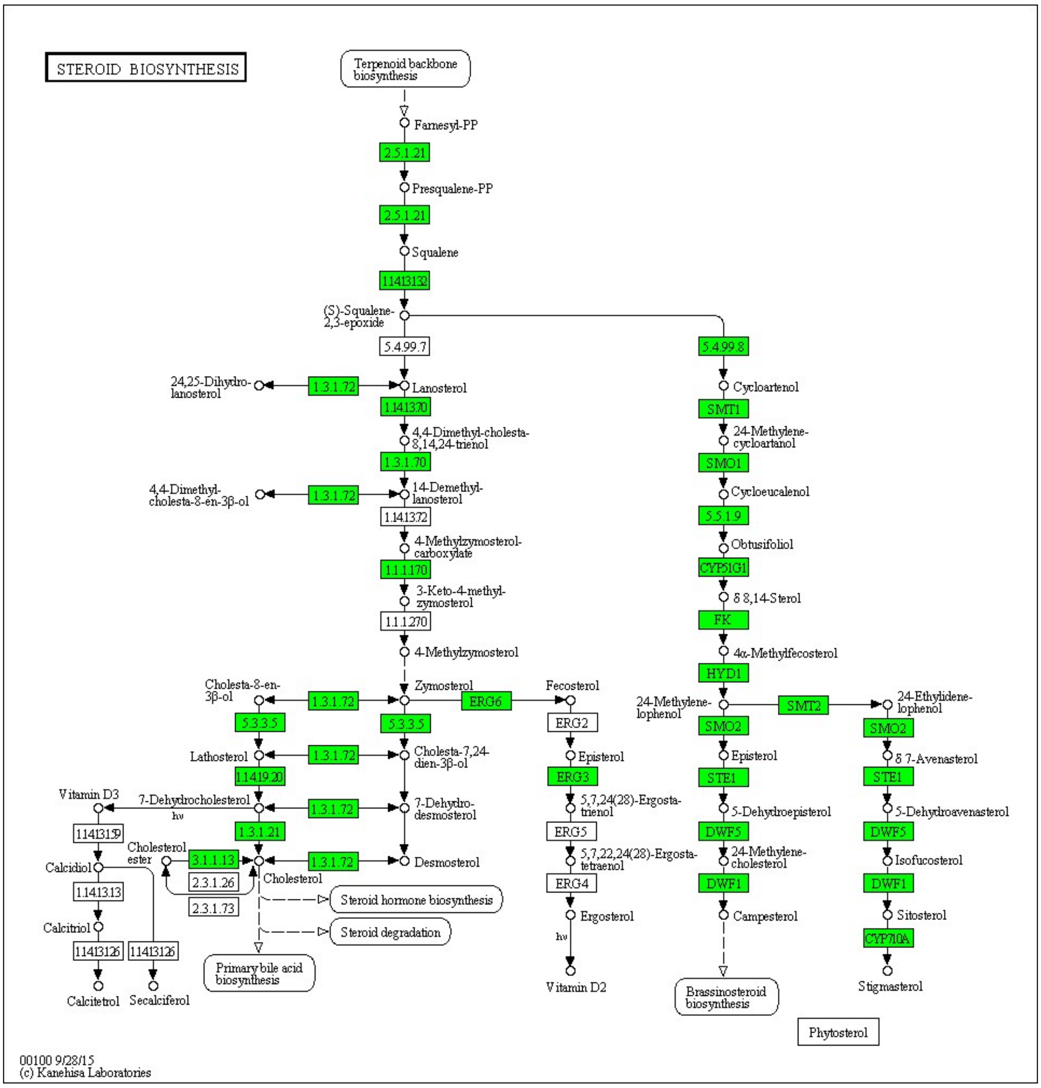

Figure S5. Map of steroid biosynthesis pathway generated by KEGG analysis. Green boxes denoted unigenes that were identified in the pathway.
